# “Everything’s a Challenge”: An Interview Study of ADHD Individuals in the Midst of the Prescription Stimulant Shortage

**DOI:** 10.1177/10870547241288351

**Published:** 2024-10-08

**Authors:** Benjamin Johnson, Daniel Stjepanovic, Janni Leung, Gary C. K. Chan

**Affiliations:** 1 National Centre for Youth Substance Use Research, The University of Queensland, Brisbane, Australia

**Keywords:** ADHD, stimulants, medication, adderall, ritalin

## Abstract

**Background:**

The shortage of prescription stimulants is an ongoing issue that is impacting the ability of individuals with ADHD to access their medication. Amidst concerns that this shortage may have a substantial impact on individuals’ ability to manage their symptoms effectively, this research seeks to understand the experiences and consequences for those affected.

**Methods:**

In this study, we interviewed individuals with ADHD who have been directly impacted by the stimulant shortage. Thematic analysis focused on identifying common themes related to challenges with medication access and the resulting effects on daily living.

**Results:**

The study uncovered significant difficulties in accessing ADHD medication due to current shortages, leading to disruptions in the management of ADHD symptoms and subsequent detriments to individuals’ professional, educational, and personal lives. Systematic controls aimed at reducing non-medical use were found to exacerbate these access issues, inadvertently compounding the challenges faced by those using medication for legitimate medical needs. Individuals also described ways they coped with the shortage, with some seeking ADHD medication via unofficial channels.

**Conclusion:**

Our findings highlight the urgency of addressing stimulant shortages to safeguard the wellbeing of individuals with ADHD. This study also calls for a critical review of policy measures regulating stimulant medication access, and their effectiveness at reducing non-medical use given the unintended consequences these regulations appear to have on individuals prescribed these medications for therapeutic purposes.

ADHD, is a neurodevelopment disorder characterized by core symptoms of inattention, hyperactivity, and impulsivity (Mercugliano, 1999). It is a condition that is prevalent globally, affecting an estimated 4% of individuals worldwide (Mohammadi et al., 2021). Originally thought only to affect children and to remit naturally prior to adulthood, ADHD symptoms have been found to extend into adulthood in 15% to 65% of cases (Adamou, 2023).

The impact of ADHD on individuals is well-documented and vast. ADHD is associated with poor outcomes such as reduced educational functioning in high-school and higher education (DuPau et al., 2018), as well as increased unemployment (Rietveld & Patel, 2019) and criminality (Engelhardt et al., 2019). Additionally, individuals with ADHD often experience relationship problems (VanderDrift et al., 2019) and elevated co-morbid mental health problems, such as anxiety and depression (Ohnishi et al., 2019) at an elevated rate compared to healthy peers. Thus, appropriate and timely treatment is paramount.

No pharmacological treatment is more effective for treating ADHD than prescription stimulants (Adamou, 2023). Stimulants work by inhibiting reuptake of dopamine and norepinephrine, increasing the concentration of these neurotransmitters in the brain (Perugi et al., 2022), which helps ADHD individuals improve concentration and focus while reducing hyperactive and impulsive behavior (Kolar et al., 2008). Treatment with stimulants have been found to significantly improve daily function (Hawk et al., 2018) and quality of life (Furczyk & Thome, 2014). Due to their proven effectiveness, prescription stimulants have become the front-line treatment for ADHD in many countries, serving as a cornerstone in managing the disorder for decades (Adamou, 2023; Kooij et al., 2019).

The stimulant shortage is an ongoing issue affecting the availability of ADHD stimulant medications. Confirmation of the shortage came in October 2022, when the U.S. Food and Drug Administration (FDA, 2022) announced a nationwide shortage of mixed amphetamine salts (trade names Adderall and Mydayis). This shortage has impacted roughly 1 in every 10 Americans who are prescribed amphetamine/dextroamphetamine reflected by a 11% decline in the estimated average monthly prescription fill rate from 2022 to 2023 (McPhillips, 2024). Additionally, other forms of prescription stimulants such as methylphenidate and lisdexamfetamine are now in short supply as well (Lovelace, 2024).

Whilst this has primarily affected the United States, an estimated 150,000 people in the U.K. are in the same position after the Department for Health and Social Care announcing a shortage of stimulants in September (Seth & Ford, 2023). Additionally, there have been current and anticipated shortages for certain ADHD stimulants in Australia as well (Therapeutic Goods Administration, 2023).

This has likely had a considerable impact on individuals with ADHD, with a survey finding that 38% of adults with ADHD have had trouble finding and filling their prescription medication over 2023 (ADDitude, 2023). The struggle is also widely reported in the media, where individuals share their experiences of severe difficulties in accessing their medications, leading to a notable decline in their daily functioning and increasing stress levels (Lovelace, 2024, Tran, 2024, Walsh, 2024). These individuals have found themselves in the role of “detectives” (Ault, 2024), in their quest to find their necessary medication.

However, while news articles have covered the issue, no academic research has yet explored the effects of the stimulant shortage on individuals. In this project, we will address this gap by conducting a qualitative study and interviewing those impacted by difficulties accessing medication in the stimulant shortage.

## Materials and Methods

### Subject Recruitment

Participants were recruited through online advertisements from January 1st to the 1st of April 2024. Advertisement methods included postings on r/ADHD, the largest English ADHD forum, and the Children and Adults with Attention-Deficit/Hyperactivity Disorder (CHADD) forum and research page. CHADD affiliate groups were also emailed to assist in the dissemination of the study within their communities.

The inclusion criteria were adult individuals experiencing difficulties accessing stimulant medication during the shortage for themselves or their child. Potential participants were encouraged to contact researchers through direct messaging on the platform, by phone, or email to schedule an online interview. An information and consent sheet, detailing study information and assurances of confidentiality and anonymity, was distributed to interested individuals.

### Sample Characteristics

Twenty participants were interviewed. Nineteen participants resided in the United States and one in Australia. Detailed demographic characteristics are presented in Table 1. We collected demographic data of the individuals directly affected by the stimulant shortage, rather than that of interviewees. For interviews with parents, the demographics of the children impacted was collected.

**Table 1.** Demographics of Individuals Impacted by Stimulant Shortage.

Characteristic (of individual impacted)	*n*
Gender	
Male	10
Female	10
Age of participant impacted (years)	
10–17	4
18–24	4
25–30	1
31–40	3
41–50	3
50–60	4
60+	1
Ethnicity	
White	10
Black or African American	9
Asian	1
Highest education level	
Currently in secondary school	4
High-school diploma	4
Bachelors degree	8
Post-graduate degree	4
Occupation	
Full-time	6
Part-time or casual	6
Self-employed	1
Not employed/working	7
City of residence	
Los Angeles	5
New York	3
Chicago	2
New Jersey	2
Silver Spring	1
Saint Paul	1
Denver	1
Pennsylvania	1
Phoenix	1
Houston	1
Sydney	1
Atlanta	1
Type of medication	
Mixed amphetamine salt (Adderall)	12
Lisdexamfetamine (Vyvanse)	4
Methylphenidate (Ritalin, Concerta)	2
Dexmethylphenidate (Focalin)	1
Methamphetamine (Desoxyn)	1

### Data Collection

Interviews were conducted between January and April 2024 via Zoom conferencing, with durations ranging from 21 to 59 min and a median length of 36 min. The interview process continued until data saturation was achieved.

Employing a semi-structured format, the interviews were guided by an interview guide (see Supplemental Appendix A), allowing deviations from the question guide to explore topics that arose naturally in conversation. The interviews commenced with the collection of verbal consent and demographic information, followed by open-ended questions regarding access to medication during the shortage, challenges arising from lack of medication access, coping mechanisms, and healthcare interactions. At the interview’s conclusion participants received a 20 USD gift card as compensation for their time.

### Data Analysis

Audio recordings were transcribed using Otter.ai, with manual checks for accuracy. The analysis began with an open coding phase where the primary researcher engaged deeply with the transcript data, identifying initial themes through a reflexive thematic analysis approach as outlined by Braun and Clarke (2006). This approach allowed for the integration of both deductive and inductive coding strategies, enabling the capture of predefined and emergent themes respectively.

Initially, multiple readings of the transcripts were conducted, marking significant passages and noting preliminary insights. Subsequent coding was carried out in stages. Firstly, provisional codes were applied to the data, reflecting both the core research questions and the new insights that emerged from the initial readings. These codes were then refined and reorganized into cohesive thematic categories.

To ensure the coding was reliable and the themes accurately represented the data, the process included several rounds of reviewing and adjusting the codes. This meant going back through the data multiple times to check if the codes were applied consistently and whether the developed themes truly captured the essence of the data. Any inconsistencies found during these reviews were corrected, and themes were adjusted as needed to better fit the data.

A final review was conducted to confirm the names and definitions of the themes. During this phase, examples that best illustrated each theme were chosen.

### Ethical Considerations

Ethical approval for this study was granted. The study emphasized ethical treatment and guideline adherence, with informed consent obtained pre-interview. Participants were assured of their right to withdraw or refuse answering questions without penalty.

Following the interviews, identifiable information and recordings were deleted to maintain anonymity. Interviewer training ensured appropriate conduct, creating a non-judgmental atmosphere, and accurate interpretation of participant responses to minimize risk and misrepresentation.

## Results

We conducted a thematic analysis to identify concurrent discussion points amongst participants in our study. Our analysis produced four themes: access to medication, treatment barriers, impact of shortage and coping strategies. The descriptions and frequencies of these themes and the recurring codes that comprised them can be seen in Table 2.

**Table 2.** Descriptions and Frequencies of Key Themes and Recurring Codes.

Theme	Description of theme	Recurring codes
Access to medication (*n* = 20)	Focuses on the direct challenge’s individuals face in accessing ADHD medications and the logistical hurdles of obtaining prescriptions.	• Pharmacies out of stocks (*n* = 20) • Calling multiple pharmacies (*n* = 16) • Driving long distances (*n* = 9) • Partial prescription fulfillment (*n* = 6) • Being switched to alternate medication (*n* = 5)
Treatment barriers (*n* = 16)	Examines systemic barriers within the healthcare system that exacerbate access difficulties.	• Lack of medication stock information (*n* = 14) • Restrictive prescription refill policies (*n* = 10) • Stigma from healthcare workers (*n* = 8)
Impact on life (*n* = 20)	Highlights the extensive impact of the medication shortage across multiple domains of individuals lives.	• Relapse of ADHD symptoms (*n* = 17) • Reduced occupational performance (*n* = 13) • Emotional instability and dysregulation (*n* = 11) • Relationship problems (*n* = 8) • Withdrawal from social activities (*n* = 4)
Coping strategies (*n* = 12)	Highlights the methods individuals employed to help overcome the impact of their lack of ADHD medication.	• Exercise (*n* = 4) • Energy drinks and alcohol as self-medication (*n* = 4) • Seeking ADHD medications via non-medical routes (*n* = 4) • Meditation (*n* = 3)

### Access to Medication

**Figure 1.**
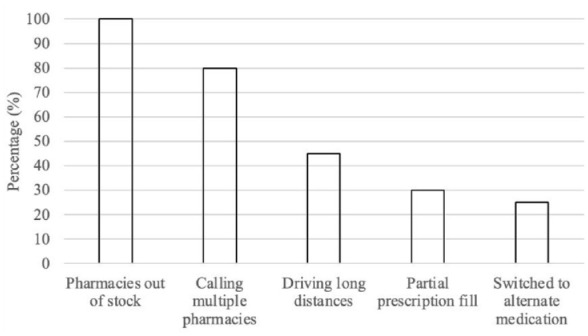
Issues with access to medications for interview participants.

Figure 1 shows the percentage of participants who encountered specific barriers when attempting to access their ADHD medication.

All participants noted a recent period of difficulty in accessing their ADHD medications, attributing this to pharmacies not having their medication in stock, typically in the past 1 to 2 years. One participant detailed their experience, stating, *“I started going to the pharmacy thinking I was picking up my medicine, only to be told, ‘We don’t have it. It’s on back order. And we don’t know when we will get more.”* In some instances, pharmacies could provide only a limited supply of medication: *“There were times when our current pharmacy could give us a few pills, like five or three, whatever they had. I then had to ask my psychiatrist to prescribe just that amount, whatever we could manage.”*

The scarcity of medication was widespread, as highlighted by another participant: *“I actually called every pharmacy in my city and the city I work in; they were all out of stock or didn’t have any stimulants at all.”*

The unpredictable availability of medications left many in a constant state of uncertainty, with one individual describing the recurring dilemma: *“Every month, I would have a new appointment and get a new prescription. During these times, there’d be like three to five days where I wouldn’t know where to get my prescription from.”*

Several participants mentioned periods during which they had to go without medication or attempt alternatives that were often less effective than their original prescriptions. *“It was very sporadic. I probably went three months with no meds. Then we started trying new ones, but a new one wouldn’t work. So, there would be another period with no medication at all,”* one participant recounted.

This shortage forced individuals to take extreme measures, such as traveling long distances in the hope of finding a pharmacy with available stock: *“At one point, I drove an hour and a half outside the city to get half of my prescription,”* shared one participant, illustrating the lengths to which some went to obtain their medication.

For the majority, this predicament remains unresolved. *“I still often have to wait because they just can’t get it sometimes when I need it. The wait is shorter now, so it’s improved, but it’s still a significant issue,”* a participant concluded, underscoring the ongoing nature of the challenge.

### Treatment Barriers

**Figure 2.**
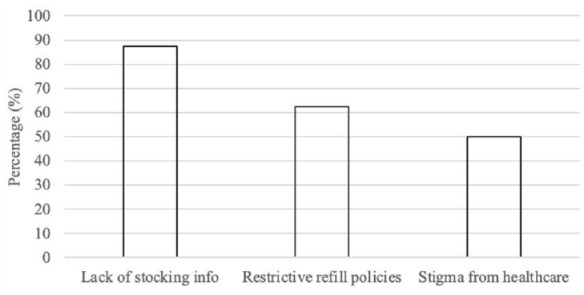
Treatment barriers for interview participants.

This theme uncovered the systemic barriers embedded within the healthcare system that intensified the hardships individuals faced. Figure 2 depicts the barriers these individuals encountered when attempting to obtain ADHD medications.

Firstly, the opaque practices of pharmacists, who often would not disclose medication stock levels, significantly hindered participants’ ability to efficiently locate necessary medications: *“for the most part, pharmacies were just like, ‘we can’t tell you whether or not we have that’. And then I would say, ‘well, how am I supposed to know whether I can get it or not?’”*. This lack of transparency forced individuals to embark on time-consuming and often fruitless visits to numerous pharmacies, seeking to find their prescriptions in stock. There was also a lack of clear communication from healthcare professionals about the reasons behind the shortage, leaving many participants in the dark and necessitating personal research to understand the crisis.

The stringent policies around prescription refills compounded the issue. As one participant explained, *“I can’t go pick it up until the day that it’s ready. . . they won’t release the medicine until it’s all gone. They won’t even give it to you like a few days early. So once it’s empty, I have to go right away to the pharmacy to get it.”* Such constraints severely limited individuals’ ability to plan ahead and manage or anticipate shortages, exacerbating the anxiety and logistical challenges posed by the medication shortage.

Additionally, many participants reported experience of stigma from healthcare professionals. As one participant shared, *“If I go to the pharmacist to get a migraine med refill, I’m treated fine. If I go to get an ADHD stimulant the attitude changes. . . . Questions like do you really need this? Is this a habit for me? People’s stigma of ADHD really came up during that time.”*

Participants believed stigma extended upwards to regulators, which contributed to the medication shortage and the restrictive policies surrounding ADHD medication. Some felt the perception of ADHD medication as non-essential or abusable had profound implications on their access to care: *“I see medication as sort of a life and death, the way somebody takes heart medicine. It’s not how some people see it, just to help somebody focus or be able to take a test. . . ADHD, is a health condition. Why does it have to have all these restrictions on it and be a controlled substance?”*

### Impact on Life

**Figure 3.**
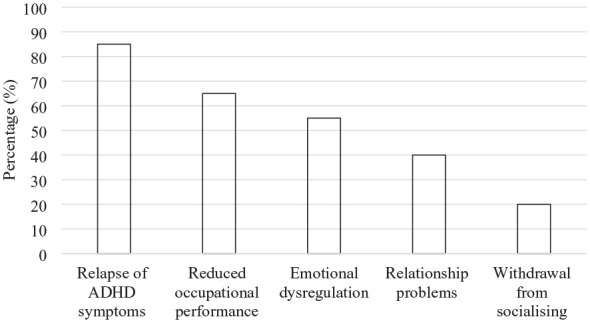
Impacts on life for interview participants.

Our study revealed the profound impact that the stimulant shortage has had on multiple domains of individuals’ lives. These are depicted in Figure 3.

Participants described a marked deterioration in daily functioning due to inconsistent medication access. The struggle to maintain routine tasks was evident, with one individual noting, *“I routinely would make wrong turns on streets or miss exits off highways. I double booked clients. I’ve lost my keys, I lost my wallet.”*

Occupational impacts were stark, from diminished job performance to the loss of employment, underscoring the critical role of medication. *“I couldn’t keep up with all the requirements of my job. I just couldn’t function in an office environment. I needed to work from home, because I had so much restless energy and would constantly get distracted”* one participant reported. Another participant noted the consequences in others who lacked access to medication: *“I [know two people] who lost their jobs and most of them were struggling badly.”*

Participants described significant challenges in managing their emotions in the absence of medication. “*I struggled with my emotional regulation. . .without medication I definitely became more irritable”* shared one participant. Some described damage to their relationships due to these difficulties: *“My kids, dad and I, we had to split. . . My emotions wouldn’t have an off switch and then I was basically all the anxiety, the depression, everything. I was kind of an uncontrolled mess.”* Additionally, some individuals reported their social life been strained: *“I cut out almost all my social things, because I was absolutely overwhelmed*.”

The shortage’s impact was particularly poignant in the context of children with ADHD. Parents recounted the distressing observations of their children’s deteriorating academic performance, emotional regulation, and social interactions. *“So summer comes around, we are experiencing shortages with the Focalin and we switched him to Concerta. It was almost immediately that his teachers began to write me and indicate that his attention was not the same”* a parent shared.

For parents, the shortage was not just a logistical nightmare but a source of profound stress and concern for their children’s safety and future. *“It took a lot of time out of my schedule to focus on this and I was really worried that he wouldn’t have it. For him, this medicine, it’s almost like it’s life or death because he can be so unsafe and so impulsive when he’s not paying attention.”*

### Coping Strategies

In response to the ADHD medication shortage, participants employed a range of coping strategies to manage their symptoms.

A couple participants sought assistance from a therapist who helped them develop coping strategies and talk through their issues. However, some individuals were frustrated at their primary care providers for the lack of support they provided during this period: *“I felt very unsupported by my doctor. . . it was as if all he could do was write a prescription and send me on my way. There was no guidance, no alternative solutions, just a prescription and wishes of good luck.”*

Incorporating physical activity into daily routines emerged as a key strategy, with one individual stating, *“Anything at all, I was doing as much exercise as I could, anything that would improve my dopamine.”* Alongside physical exercise, behavioral mechanisms such as meditation, the use of timers and white noise were mentioned: *“To focus I would put on white noise and was tried to kind of power through things.”*

The consumption of energy drinks was another prevalent coping mechanism among participants. The stimulant effects of caffeine were sought after as a temporary substitute for prescription medications. An individual recounted, “*I was drinking energy drinks, like three a day, like six to eight Diet Cokes a day*.” Additionally, a couple of participants also described self-medicating using alcohol: *“I just basically went back to like that not so functioning alcoholic. . . the alcohol gave me the ability to focus and calm down my nervous system in my head enough to function.”*

Faced with persistent shortages and the inability to access prescribed medications, some participants resorted to acquiring stimulants through non-medical channels. One participant shared their experience: *“I got my medication unofficially, because pharmacies didn’t have and they had, it was a lot more expensive. . . I got it through dealers that I knew that sold it.”*

## Discussion

The current literature on the stimulant medication shortage is scarce. Our study provides critical, novel insights into the issue’s breadth and depth, highlighting its significant impact on individuals with ADHD.

The sudden cessation of these crucial medications was found to have considerable impacts to individuals ADHD symptoms and abilities to function. This is unsurprising considering the importance of prescription stimulants for many individuals with ADHD ability to function. ADHD medications have been shown to effectively manage symptoms in children (Mechler et al., 2022) and adults (Boesen et al., 2022) alike. Additionally, they have also been found to improve long-term outcomes, reducing the prevalence of mood disorders, substance use, criminality, suicidality, and enhancing academic and occupational achievements (Boland et al., 2020).

This shortage not only complicates the management of ADHD symptoms but also significantly disrupts the lives of those affected, as our findings suggest. Individuals frequently face the difficult choice of discontinuing their medication, adversely affecting their education, employment, mental health, and overall quality of life. Given the substantial impact that this shortage is having on individual’s wellbeing and functioning, it is of vital importance that such shortages are resolved promptly. Future research should quantitatively estimate the number of individuals affected by the shortage, to understand the full scope of impact this shortage is likely to have resulted in.

Participants described how the current healthcare system exacerbates access difficulties. These systems, designed to curb non-medical use (BMC Medicine, 2023) inadvertently impose significant burdens on legitimate medical usage. An easing of restrictions should be considered for the sake of individuals with ADHD. Revising policies to allow flexibility in medication pickup and refill procedures can significantly alleviate the burden on patients experiencing shortages. We must consider how effective these policies and the lack of transparency are at reducing non-medical, illegitimate use considering the severe impact it has on the ability of individuals with ADHD to access appropriate care.

In exploring adaptive strategies employed by individuals with ADHD, our investigation aligns with existing research that highlights the utilization of various non-pharmacological methods, such as the consumption of caffeine, the use of timers (Canela et al., 2017) and exercise, which has been found to be an effective for reducing ADHD symptoms (Gapin et al., 2011). A salient concern emerging from our study is the reported inadequacy of support from healthcare providers, extending beyond the facilitation of medication access. The findings emphasize the necessity for clinicians to be proficient in a repertoire of management strategies, including behavioral therapy to adequately address the multifaceted needs of individuals with ADHD, especially during times of stimulant medication scarcity.

Additionally, we found some individuals reported obtaining stimulant medication illegally. The difficulty in accessing ADHD medications legally may paradoxically not deter but rather drive individuals toward seeking medications through illegitimate means, suggesting current strategies might be counterproductive. Regulators should be aware of this potential effect when making decisions regarding the availability of these medications.

While our study primarily focuses on the experiences of those in the United States, it is crucial to recognize that the issue may have broader implications, considering most manufacturers are based in the U.S. (360 Research Reports, 2022). Understanding the impact of stimulant medication shortages in other countries is essential for a comprehensive understanding of the scope of this problem. Furthermore, the study’s concentration on urban populations could overshadow the distinct challenges faced by individuals in rural or remote settings. Such locations may have limited pharmacy options, which could intensify the difficulties in accessing ADHD medications.

Our results cannot be generalized to indicate what percentage of all ADHD patients has been affected. To bridge the gap in quantitative data and build upon our findings, future research should focus on larger-scale studies that quantify the extent of the stimulant shortage’s impact on the broader ADHD population. Such research is essential for understanding the full scope of the issue and informing policy decisions to mitigate similar challenges in the future.

Future research should also consider the impact on prescribers, who may face increased pressure and ethical dilemmas in managing their patients’ needs during shortages. Additionally, the economic implications, such as cost inflation of these medications (Arielle Bosworth et al., 2023), need to be explored to understand the broader financial impact on both healthcare systems and patients.

## Limitations

The analysis was conducted by a single coder, which may introduce interpretive bias, as personal biases could influence the selection and interpretation of themes. Additionally, without multiple coders, there’s a risk of inconsistent code application across the dataset. To address these concerns, an iterative review process was employed, and a detailed audit trail was maintained. While measures were taken to mitigate these risks through rigorous methodological adherence, the findings should be interpreted with an understanding of this limitation

Additionally, our study did not distinguish between short-acting and long-acting stimulant medications, which may have different implications for treatment and accessibility. This lack of differentiation means our findings may not fully reflect the varied impacts of medication shortages on individuals using different types of ADHD medications. Future studies should consider these distinctions to provide a more comprehensive understanding of the effects of medication shortages.

## Conclusions

Barriers to obtaining prescription stimulants significantly affect various aspects of life for individuals with ADHD. Efforts to deter non-medical use have inadvertently made it more difficult for those who need these medications for legitimate health reasons to obtain them. These observations highlight the pressing necessity to rectify current shortages to protect the well-being of people with ADHD. Additionally, there is an essential need to reassess the efficacy of current policies to ensure they effectively reduce non-medical use without hindering necessary medical access.

## Supplemental Material

sj-docx-1-jad-10.1177_10870547241288351 – Supplemental material for “Everything’s a Challenge”: An Interview Study of ADHD Individuals in the Midst of the Prescription Stimulant Shortage Supplemental material, sj-docx-1-jad-10.1177_10870547241288351 for “Everything’s a Challenge”: An Interview Study of ADHD Individuals in the Midst of the Prescription Stimulant Shortage by Benjamin Johnson, Daniel Stjepanovic, Janni Leung and Gary C. K. Chan in Journal of Attention Disorders
