# Supplementary material for: “Everything’s a Challenge”: An Interview Study of ADHD Individuals in the Midst of the Prescription Stimulant Shortage
Source: J Atten Disord. 2024 Oct 8;29(2):120–7. doi: 10.1177/10870547241288351 (PMC11585964; doi:10.1177/10870547241288351)
Supplement: sj-docx-1-jad-10.1177_10870547241288351 – Supplemental material for “Everything’s a Challenge”: An Interview Study of ADHD Individuals in the Midst of the Prescription Stimulant Shortage [file sj-docx-1-jad-10.1177_10870547241288351.docx]

**APPENDIX A: Question Guide for Semi-Structured Interview**

**Screening Questions:**

1. What specific ADHD medication(s) have you been prescribed? Please list them.
2. What benefits do you receive from your ADHD medication(s).
3. How regularly do you take your ADHD medication (e.g., daily, only on weekdays, as needed)?
4. Have you experienced difficulties in accessing your ADHD medication? If yes, can you briefly describe these challenges (e.g., pharmacy shortages, insurance issues, prescription delays)?

**Demographics:**

1. How old are you?
2. What is your gender identity?
3. Are you currently employed, and if so, what is your occupation?
4. Are you currently studying? Could you talk about your education history and any qualifications you have?

**Access to Medication:**

1. What stimulant medications are you currently prescribed or have used in the past?
2. Have you experienced any difficulties in obtaining your prescribed medication during the recent shortage?
3. If you experienced difficulties, how did you manage or cope with the situation?
4. Were you offered any alternative medications, and how did they compare to your regular prescription?

**Impact on Life:**

1. Can you describe how the stimulant shortage has affected your daily life?
2. Do you believe the shortage has had an impact on your physical or mental health?
3. Have you noticed any changes in your social interactions or professional life due to the shortage?

**Coping and Support:**

1. What strategies have been most effective in dealing with the impact of the shortage?
2. Have you sought support from any community groups, online forums, or other networks during the shortage?
3. What coping strategies have you found most helpful?

**Healthcare Interaction:**

1. How satisfied have you been with the communication and support from your healthcare providers during the stimulant shortage?
2. What, if anything, do you wish had been handled differently by your healthcare providers?

**Perceptions and Future Concerns:**

1. How has the stimulant shortage affected your perception of the healthcare system?
2. Do you have concerns about future medication shortages, and how are you planning to address them?

**Miscellaneous:**

1. Do you have any suggestions for healthcare providers or policymakers on how to manage medication shortages effectively?
2. Would you like to share any additional experiences or stories related to the stimulant shortage?
3. How has this experience influenced your views on medication and health management in general?

“Thank you for sharing your valuable insights. Is there anything else you’d like to add before we conclude?”
